# Traineeships at the European Centre for Disease Prevention and Control (ECDC)

**DOI:** 10.2807/1560-7917.ES.2024.29.39.2409268

**Published:** 2024-09-26

**Authors:** Eurosurveillance editorial team

**Affiliations:** 1 European Centre for Disease Prevention and Control (ECDC), Stockholm, Sweden

The European Centre for Disease Prevention and Control (ECDC) invites applications for the 2025 Traineeship Programme.

The application deadline expires on 21 Oct 2024.

For more information, please visit the ECDC website: https://www.ecdc.europa.eu/en/about-ecdc/work-ecdc/traineeships

